# From Tradition to Innovation: Acupuncture Therapies Targeting Ferroptosis in Neurological Disorders

**DOI:** 10.1002/brb3.70827

**Published:** 2025-09-02

**Authors:** Haiyan Sun, Haiqing Qian, Heng Qian, Min Qian

**Affiliations:** ^1^ Translational Medical Innovation Center Zhangjiagang TCM Hospital Affiliated to Nanjing University of Chinese Medicine Zhangjiagang Jiangsu People's Republic of China; ^2^ Department of Anesthesiology Zhangjiagang TCM Hospital Affiliated to Nanjing University of Chinese Medicine Zhangjiagang Jiangsu People's Republic of China; ^3^ Department of Reproduction Zhangjiagang TCM Hospital Affiliated to Nanjing University of Chinese Medicine Zhangjiagang Jiangsu People's Republic of China

**Keywords:** acupuncture, ferroptosis, neurodegenerative diseases, stroke

## Abstract

**Introduction:**

Age‐related neurodegenerative diseases (NDDs) and stroke represent significant public health challenges due to their high morbidity, mortality, and associated cognitive decline. Current therapeutic strategies often fall short in addressing the complex underlying pathological mechanisms, underscoring the urgent need for innovative treatment approaches. Ferroptosis, an iron‐dependent form of regulated cell death driven by lipid peroxidation, has emerged as a critical factor contributing to neuronal damage in NDDs and stroke.

**Methods:**

To explore how acupuncture‐related therapies may modulate ferroptosis and contribute to neuroprotection, we conducted a comprehensive narrative review. A systematic search was performed in PubMed using targeted keywords including “ferroptosis,” “interventions” (such as acupuncture, electroacupuncture, and moxibustion), and “diseases” (including age‐related neurodegenerative diseases and stroke). The search was restricted to articles published before April 2025 with full‐text availability in English.

**Results:**

This review consolidates recent findings on the potential of acupuncture‐related therapies to target ferroptosis in NDDs and stroke. Traditional acupuncture, electroacupuncture (EA), and moxibustion have shown promising neuroprotective effects through multiple mechanisms. Emerging evidence suggests that these interventions can regulate ferroptosis by modulating key molecular pathways involved in iron metabolism, lipid peroxidation, and oxidative stress. These actions are thought to alleviate synaptic dysfunction and slow cognitive decline—processes where ferroptosis plays a central role. Such insights enhance our understanding of how traditional Chinese medicine could influence cognitive outcomes in age‐related NDDs and stroke.

**Conclusion:**

Understanding the role of ferroptosis in NDDs and stroke is essential for developing novel therapeutics. Based on current findings, this review proposes future research directions aimed at integrating acupuncture into modern neurotherapeutic frameworks. Combining traditional medicine with contemporary medical approaches offers new opportunities for promoting brain health and improving patient outcomes.

## Graphical abstract

• *Ferroptosis drives cognitive decline in stroke and age‐related NDDs*

 Iron‐dependent lipid peroxidation and oxidative stress trigger ferroptosis, a central mechanism underlying neuronal injury and cognitive deterioration in stroke and age‐related NDDs.

• *Acupuncture‐related therapies target ferroptosis through multifaceted regulation*

 Acupuncture, electroacupuncture, and moxibustion regulate key ferroptotic pathways, including iron homeostasis, antioxidant defense, and lipid peroxidation, offering multitarget neuroprotection.

• *Integrating tradition with modern neuroscience for targeted therapy*

 This review integrates acupuncture‐related therapies with ferroptosis biology, establishing a scientific framework for developing innovative, mechanism‐based interventions for stroke and age‐related NDDs.

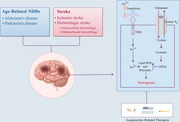

**Abbreviations**
15‐LOX: 15‐lipoxygenase
6‐OHDA: 6‐hydroxydopamine
ACSL4: acyl‐CoA synthetase long‐chain family member 4
AD: Alzheimer's disease
APP/PS1: amyloid precursor protein/presenilin 1
ATP: adenosine triphosphate
Aβ: β‐amyloid
COX‐2: cyclooxygenase‐2
DFO: deferoxamine
DMT1: divalent metal transporter 1
DTI: diffusion tensor imaging
EA: electroacupuncture
fMRI: functional magnetic resonance imaging
FTH1: ferritin heavy chain 1
GPX4: glutathione peroxidase 4
GSH: glutathione
GSSG: glutathione disulfide
HS: hemorrhagic stroke
I/R: ischemia‐reperfusion
ICH: intracerebral hemorrhage
IS: ischemic stroke
Keap1: Kelch‐like ECH‐associated protein 1
LIP: labile iron pool
LOX: lipoxygenase
LPCAT3: lysophosphatidylcholine acyltransferase 3
MCAO: middle cerebral artery occlusion
MDA: malondialdehyde
miRNA: microRNA
mTOR: mechanistic target of rapamycin
NCOA4: nuclear receptor coactivator 4
NDD: neurodegenerative disease
NFT: neurofibrillary tangle
NRF2: nuclear factor erythroid 2‐related factor 2
PD: Parkinson's disease
PET: positron emission tomography
PTGS2: prostaglandin‐endoperoxide synthase 2
PUFA‐PL
: polyunsaturated fatty acid phospholipid
PUFA: polyunsaturated fatty acid
ROS: reactive oxygen species
SA: scalp acupuncture
SAH: subarachnoid hemorrhage
SLC7A11: solute carrier family 7 member 11
SNpc: substantia nigra pars compacta
SNX5: sorting nexin 5
SREBP1: sterol regulatory element‐binding protein 1
STEAP3: six‐transmembrane antigen of prostate 3
TCM: traditional Chinese medicine
TF: transferrin
TFR1: transferrin receptor 1
TH: tyrosine hydroxylase

## 1 Introduction

The incidence of neurological disorders, particularly age‐related neurodegenerative diseases (NDDs) and stroke, has escalated to concerning levels, posing considerable challenges to healthcare systems (Ben‐Shlomo et al. 2024; Parry‐Jones et al. 2025). The prevalence of these conditions is rising in tandem with aging populations, leading to profound socioeconomic consequences. Current pharmacological interventions, while offering symptomatic relief, frequently exhibit limited efficacy against underlying pathological processes and undesirable side effect profiles (Carbone and Djamshidian 2024; Imbimbo et al. 2025). This therapeutic impasse has intensified research into alternative approaches targeting fundamental disease mechanisms with improved safety characteristics.

The identification of ferroptosis as a distinct regulated cell death pathway has transformed our understanding of neurological pathogenesis. This iron‐dependent process, characterized by lipid peroxidation and antioxidant system failure, plays a pivotal role in both acute neural injury and chronic neurodegeneration (X. Hu, Bao, et al. 2024; Streit et al. 2025). Substantial evidence implicates ferroptosis in neuroinflammatory responses, synaptic impairment, and subsequent cognitive deterioration through complex molecular interactions (Z. Liu, Shen, et al. 2025; Song et al. 2025). These findings not only advance mechanistic comprehension but also establish ferroptosis as a viable therapeutic target.

Acupuncture‐related therapies—encompassing traditional acupuncture, electroacupuncture (EA), and moxibustion—have emerged as promising interventions for neuroprotection (Fu et al. 2024; Z. Zhang, Chen, et al. 2023). Grounded in traditional Chinese medicine (TCM) yet compatible with modern scientific methodologies, these techniques represent a unique synthesis of empirical knowledge and contemporary medicine. Emerging data suggest acupuncture‐related therapies’ benefits may derive from their multifaceted influence on iron homeostasis, oxidative stress regulation, and inflammatory modulation—all critical mechanisms of ferroptosis (Fu et al. 2024; Z. Zhou, Yu, et al. 2024).

This review provides a comprehensive biological evaluation of acupuncture's therapeutic effects on ferroptosis in neurological disorders, particularly age‐related NDDs and stroke. We systematically analyze experimental evidence for acupuncture's anti‐ferroptosis, elucidate its underlying molecular mechanisms, and critically assess current research limitations and translational potential. By integrating traditional acupuncture practice with modern ferroptosis biology, this work establishes a scientific framework for developing targeted interventions against ferroptosis‐mediated neural damage while bridging empirical medical knowledge with contemporary neurobiological understanding.

## 2 Overview of Ferroptosis

In 2008, Yang and Stockwell (2008) identified a novel form of cell death during their screening of antitumor agents, distinct from apoptosis, necrosis, and autophagy. This iron‐dependent mechanism was later named “ferroptosis” by Dixon et al. (2012). Ferroptosis is characterized by the accumulation of free iron within tissues, which catalyzes hydroxyl radical production via the Fenton reaction, driving lipid peroxidation in cell membranes. Simultaneously, glutathione (GSH) levels and glutathione peroxidase 4 (GPX4) activity are diminished, impairing the detoxification of lipid peroxides. The resulting oxidative stress and lipid peroxide accumulation lead to irreversible damage and cell death (J. Li et al. 2020).

**FIGURE 1:**
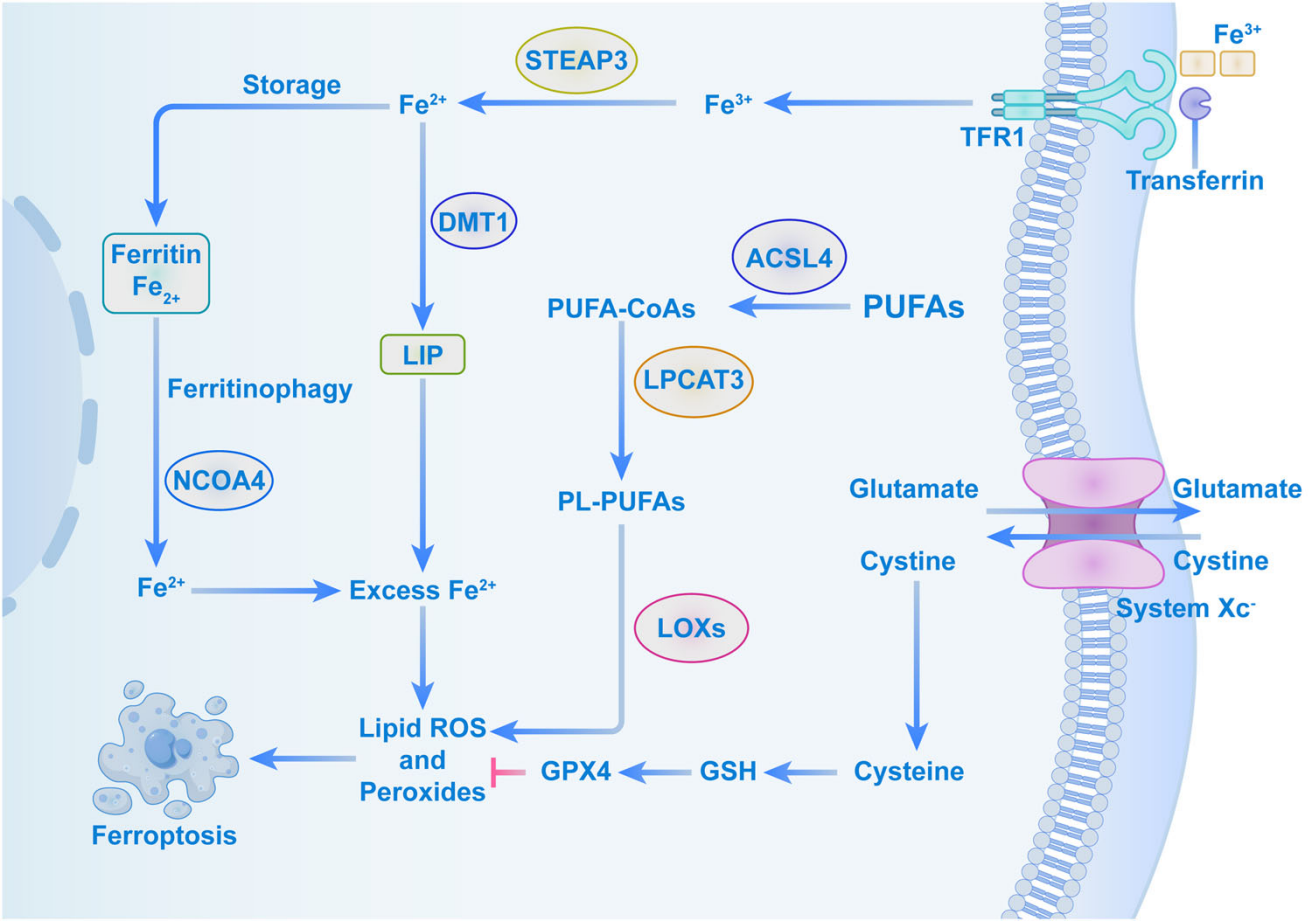
The mechanism of ferroptosis. Fe^3+^ is transported in the bloodstream by TF as TF‐Fe^3+^. This complex enters cells via endocytosis after binding to TFR1. Inside the cell, Fe^3+^ is released from TF and reduced to Fe^2+^ by STEAP3. Fe^2+^ is then either stored as ferritin or moved to the LIP via DMT1. Ferritin is degraded to release free iron through ferritinophagy, mediated by NCOA4. Excess Fe^2+^ can cause lipid peroxidation via the Fenton reaction and iron‐dependent oxidase, leading to potential damage. In amino acid metabolism, extracellular cystine and intracellular glutamate are exchanged at a 1:1 ratio through System Xc‑ on the cell membrane. Cystine is converted into GSH, a powerful antioxidant that reduces ROS. GPX4 maintains GSH's reducibility. In ferroptosis, lipid peroxidation is crucial, with PUFAs playing a key role. ACSL4 converts PUFAs to PUFA‐CoAs, which are esterified by LPCAT3 into PL‐PUFAs. These are oxidized by LOXs into lipid hydroperoxides. In the presence of Fe^2+^, these hydroperoxides become harmful lipid radicals, causing cellular damage and leading to ferroptosis.

Morphologically, ferroptosis is marked by distinct ultrastructural changes, including disrupted cell membrane integrity, cytoplasmic swelling, chromatin condensation, mitochondrial marginalization, reduced or absent mitochondrial cristae, increased mitochondrial membrane density, and eventual rupture of the outer mitochondrial membrane (Kong et al. 2023). Biochemically, ferroptosis is defined by GSH depletion, reduced GPX4 activity, and iron overload, which collectively promote lipid peroxidation and the accumulation of phospholipids containing polyunsaturated fatty acids (PUFA‐PLs) (X. D. Zhang, Liu, et al. 2023). The regulatory framework of ferroptosis encompasses complex metabolic and signaling networks, primarily involving dysregulated iron metabolism, imbalances in antioxidant systems, and excessive lipid peroxidation (Figure 1).

## 3 Materials and Methods

### 3.1 Comprehensive Search Strategy

To ensure a thorough and systematic collection of relevant literature, we conducted an extensive search in the PubMed database using a combination of targeted keywords, including “ferroptosis,” “interventions” (including acupuncture, electroacupuncture, and moxibustion), and “diseases” (including age‐related neurodegenerative diseases and stroke). The search included all articles published prior to April 2025, with full‐text availability. This approach identified a substantial body of research investigating the effects of acupuncture‐based therapies on ferroptosis inhibition in the treatment of age‐related NDDs and stroke.

### 3.2 Inclusion and Exclusion Criteria

To ensure methodological rigor and relevance, we applied strict inclusion and exclusion criteria during the literature screening process. Included studies were required to meet the following criteria: (1) original peer‐reviewed research articles (excluding reviews, case reports, editorials, and conference abstracts); (2) interventions primarily involving acupuncture, EA, or moxibustion; (3) investigation of ferroptosis‐related pathways in in vivo animal models of age‐related neurodegenerative diseases or stroke; and (4) availability of full text in English.

Excluded studies included those that (1) did not directly investigate ferroptosis or oxidative stress mechanisms; (2) relied solely on in vitro or clinical data without in vivo validation; (3) focused on non‐acupuncture interventions such as herbal medicine or pharmacological agents; (4) lacked clear methodologies or reproducible experimental designs; or (5) were non‐English publications or preprints without peer review. Additionally, studies with small sample sizes (*n* < 3 per group) were excluded unless they provided novel mechanistic insights underrepresented in the current literature. Table 1 provides a summary of the most recent studies, highlighting the therapeutic effects of these interventions and the underlying molecular mechanisms related to ferroptosis modulation.

**TABLE 1:** Summary of recent studies on acupuncture‐related therapies for age‐related NDDs and stroke.

Model	Acupuncture type/frequency/time	Acupoint	Major efficacy	Reference
MCAO model	EA/2 or 100 Hz/30 min 7 days	Baihui (GV20), Shuigou (GV26), bilateral Sanyinjiao (SP6), bilateral Neiguan (PC6)	inhibit ferroptosis by regulating oxidative stress and iron‐related proteins	G. Li et al. 2021
MCAO model	EA/2 or 15 Hz/30 min 6 days	Baihui (GV20), Zusanli (ST36)	reduce oxidative stress by regulating iron transport‐related proteins	Liang et al. 2021
MCAO model	EA/3.85 or 6.25 HZ/30 min 3 days	Renzhong (DU26), Baihui (DU20)	reduce ROS accumulation and inhibit ferroptosis	G. L. Wang et al. 2023
MCAO model	EA /2 or 15 Hz/ 20 min 7 days	Baihui (GV20), Fengfu (GV16), Dazhui (GV14)	inhibit ferroptosis by increased GPX4 and decreased ACSL4, TFR, 15‐LOX, COX‐2	X. Q. Wu et al. 2023
MCAO model	EA /2 or 15 Hz/ none	Baihui (GV20)	inhibit oxidative stress and ferroptosis	Ye et al. 2024
MCAO model	EA/2 or 15 Hz/40 min 3 days	Shuigou (GV26), Neiguan (PC6), Sanyinjiao (SP6)	inhibit ferroptosis by regulating oxidative stress and iron metabolism	Q. Wang et al. 2024
MCAO model	EA/2 Hz/15 min 2 weeks	Fengchi (GB20), Fengfu (DU16), and Dazhui (GV14)	inhibit oxidative stress and ferroptosis by activating mTOR/SREBP1 pathway	Lang et al. 2024
MCAO model	Acupuncture/none/30 min	Neiguan (PC6), Shuigou (GV26)	inhibits NCOA4‐mediated ferritinophagy	Xinchang et al. 2024
MCAO model	EA/2 or 100 Hz/30 min 7 days	Neiguan (PC6),Shuigou(DU26),Sanyinjiao (SP6), Baihui(DU20)	inhibit ferroptosis by activating Nrf2 and upregulating SLC7A11 and GPX4	Zhu et al. 2024
MCAO model	EA/2 or 15 Hz/30 min 3 days	Baihui (GV20), Dazhui (GV14), Shuigou (GV26)	inhibit inflammation, oxidative stress, and ferroptosis by activating Nrf2 pathway	Pu et al. 2025
MCAO model	EA/1 or 20 Hz/30 min	LI11 and ST36	inhibit ferroptosis by suppressing KAT3B‐induced stabilization of ACSL4	F. Liu, Chen, et al. 2025
MCAO model	Moxibustion/none/30 min 7 days	Baihui (GV20), Dazhui (GV14)	inhibit ferroptosis by regulating GSH metabolism, lipid metabolism, and iron metabolism	J. Zhang, Cai, et al. 2023
ICH model	Acupuncture/none/30 min 3 days	Baihui (DU20)‐penetrating‐Qubin (GB7)	reduce neuronal cell death, inflammation, and ferroptosis by targeting miR‐23a‐3p/NFE2L2 pathway	Kong et al. 2021
ICH model	scalp acupuncture/none/30 min 7 days	Baihui (DU20)‐penetrating‐Qubin (GB7)	inhibit ferroptosis by activating the antioxidant pathway, specifically the p62/Keap1/Nrf2 axis, and upregulating FTH1 and GPX4	M. Y. Li et al. 2022
ICH model	EA/2 or 15 Hz/30 min 14 days	Baihui (GV20), Dazhui (GV14)	exert cerebral protective effects by reducing Hepc expression and promoting iron metabolism	Q. Chen et al. 2022
APP/PS1 model	EA/2 Hz/15 min 28 days	Baihui (GV20), Yintang (GV29), Shuigou (GV26)	inhibit ferroptosis by activating p62/Keap1/Nrf2 pathway	Y. Chen et al. 2024
APP/PS1 model	EA/2 or 100 Hz/20 min 28 days	Baihui (GV20), Yintang (EX‐HN3), Shuigou (GV26)	inhibit ferroptosis by upregulating of GPX4 and the downregulating of PTGS2	Y. T. Li et al. 2023
Oral administration of rotenone model	EA/2 or 15 Hz/20 min 14 days	Baihui (GV20), Quchi (LI11), Zusanli (ST36)	Protect dopaminergic neurons by regulating oxidative stress and cell apoptosis induced by ferroptosis	Ma et al. 2023
6‐OHDA‐induced model	Moxibustion/none/20 mins 6 weeks.	Baihui (GV20), Sishencong (EX‐HN1)	inhibit ferroptosis by raising GPX4 and FTH1, and increasing the number of TH‐positive cells in SN	Lu et al. 2019
6‐OHDA‐induced model	Moxibustion/none/30mins 4 weeks.	Baihui (GV20)	alleviate the damage to dopamine neurons by suppressing ferroptosis	Z. Huang, Si, et al. 2021
6‐OHDA‐induced model	Moxibustion/none/20mins 4 weeks.	Baihui (GV20), Sishencong (EX‐HN1)	inhibit ferroptosis by downregulating SNX5, promoting GSH, decreasing Fe^2+^ and MDA, upregulating the ratio of GSH/GSSG and the expression of GPX4 and FTH1 in the corpus striatum	D. Gao et al. 2024

## 4 Anti‐Ferroptosis Effect of Acupuncture‐Related Therapies in Stroke

Stroke, clinically known as cerebrovascular accident, poses a significant threat to global health due to its high morbidity and mortality rates (Prust et al. 2024). As a complex neurological disorder, stroke manifests primarily in two distinct forms: ischemic stroke (IS) resulting from cerebral blood flow obstruction, and hemorrhagic stroke (HS) caused by cerebral vessel rupture. Contemporary research has elucidated that post‐stroke neuronal injury involves intricate pathological mechanisms, with ferroptosis emerging as a crucial contributor to secondary brain damage (Y. Liu, Fang, et al. 2022). This iron‐dependent form of regulated cell death is characterized by excessive lipid peroxidation and disruption of cellular antioxidant defenses, processes that are particularly detrimental in the brain's iron‐rich environment (G. Gao et al. 2023).

The brain's unique metabolic characteristics render it exceptionally vulnerable to ferroptosis (Long et al. 2023). As the organ with the highest oxygen consumption and lipid content in the human body, neural tissue maintains a delicate redox balance that can be readily disrupted by ischemic or hemorrhagic insults. Iron, while essential for adenosine triphosphate (ATP) production and neurotransmitter synthesis, becomes pathological when homeostasis is disturbed, catalyzing the formation of reactive oxygen species (ROS) through Fenton chemistry. This oxidative stress leads to the peroxidation of PUFA‐PLs in neuronal membranes, ultimately triggering ferroptosis (S. Zhang, Xin, et al. 2022).

### 4.1 Mechanisms of Acupuncture‐Related Therapies in Modulating Ferroptosis in IS

IS, caused by a sudden cessation of cerebral blood flow, is a leading cause of neurological dysfunction and disability. While pharmacological thrombolysis remains an effective treatment, its clinical application is limited by a narrow therapeutic window and the risk of exacerbating ischemia‐reperfusion (I/R) injury following reperfusion (K. Liu, Wang, et al. 2025). These limitations have driven interest in complementary therapies, with acupuncture‐related interventions gaining attention for their potential neuroprotective effects (H. Jiang et al. 2024). Increasing evidence suggests that acupuncture‐related therapies can mitigate secondary brain damage after stroke, particularly by modulating ferroptosis (C. Wu et al. 2025), a key pathological mechanism in I/R injury.

Ferroptosis, a form of regulated cell death driven by iron‐dependent lipid peroxidation, is a critical contributor to neuronal injury in cerebral I/R (L. Zhang et al. 2024). This process disrupts membrane integrity, exacerbates oxidative stress, and amplifies neuronal damage. Acupuncture‐related therapies have demonstrated the ability to regulate ferroptosis through multiple mechanisms, targeting oxidative stress, iron homeostasis, and lipid metabolism (Z. Zhou, Yu, et al. 2024). Studies have shown that acupuncture at specific acupoints, including Baihui (GV20), Shuigou (GV26), Sanyinjiao (SP6), and Neiguan (PC6), exerts neuroprotective effects by modulating ferroptosis‐related pathways (G. Li et al. 2021). These effects include the upregulation of antioxidant systems, such as GPX4 and GSH, and the downregulation of iron transport proteins, including transferrin (TF) and transferrin receptor (TFR). Additionally, acupuncture has been found to inhibit nuclear receptor coactivator 4 (NCOA4)‐mediated ferritinophagy, a process that degrades ferritin and releases iron into lysosomes (Xinchang et al. 2024). By suppressing ferritinophagy, acupuncture helps maintain iron homeostasis and reduces oxidative stress, thereby mitigating ferroptosis. Collectively, these findings highlight the ability of acupuncture to modulate ferroptosis through diverse mechanisms, offering a novel approach to reducing neuronal damage in cerebral I/R injury.

EA, which integrates electrical stimulation with traditional acupuncture techniques, further enhances these neuroprotective effects through distinct molecular mechanisms. Research has shown that EA at Renzhong (GV26) and Baihui (GV20) significantly improves neurological outcomes and reduces infarct volume by decreasing ROS accumulation and inhibiting ferroptosis (G. L. Wang et al. 2023). Additional studies reveal that multi‐acupoint EA stimulation at Baihui (GV20), Fengfu (GV16), and Dazhui (GV14) prior to injury mitigates neurological deficits through coordinated regulation of ferroptosis markers, including increased GPX4 expression and decreased levels of acyl‐CoA synthetase long‐chain family member 4 (ACSL4), TFR, 15‐lipoxygenase (15‐LOX), and cyclooxygenase‐2 (COX‐2) (X. Q. Wu et al. 2023). Similarly, EA applied to other acupoint combinations, such as Shuigou (GV26), Neiguan (PC6), and Sanyinjiao (SP6), has demonstrated comparable neuroprotective effects (Q. Wang et al. 2024). In addition, EA has shown superior efficacy over deferoxamine (DFO) in reducing oxidative stress‐mediated neuronal damage, highlighting its potential as a more comprehensive therapeutic intervention (Q. Wang et al. 2024). Notably, pretreatment with EA at Baihui (GV20) combined with Zusanli (ST36) demonstrated extended neuroprotection spanning both acute and subacute phases of I/R injury (Liang et al. 2021). Importantly, EA pretreatment at Baihui (GV20) alone also significantly reduced cerebral infarction volume through dual mechanisms of ferroptosis inhibition and oxidative stress attenuation (Ye et al. 2024). These complementary findings establish EA's dual therapeutic modality—serving as both an acute neuroprotective intervention and a preventive strategy.

At the molecular level, EA provides neuroprotection by activating the mechanistic target of rapamycin/sterol regulatory element‐binding protein 1 (mTOR/SREBP1) signaling pathway, which is crucial for regulating cell growth, metabolism, and stress responses (Lang et al. 2024). SREBP1, as a downstream element of mTOR, regulates lipid and glucose metabolism and contributes to ferroptosis inhibition (Y. Li et al. 2024). Furthermore, EA activates the nuclear factor erythroid 2‐related factor 2 (NRF2) pathway, upregulating solute carrier family 7 member 11 (SLC7A11) and GPX4 expression, thereby enhancing antioxidant defenses and suppressing ferroptosis (Zhu et al. 2024). Consistent with this mechanism, Pu et al. (2025) demonstrated that EA pretreatment effectively mitigates inflammation and oxidative stress while inhibiting ferroptosis via Nrf2 activation, ultimately delaying the progression of IS. EA has also been shown to reduce lysine acetyltransferase 3B (KAT3B) expression, decreasing succinylation‐mediated ACSL4 stability (F. Liu, Chen, et al. 2025). Since lysine succinylation is a marker of metabolic crises in ischemic tissues (Lian et al. 2024), EA's ability to modulate this process may further enhance its neuroprotective efficacy.

Moxibustion, another acupuncture‐related therapy, complements these approaches through thermal stimulation of acupoints. J. Zhang, Cai, et al. (2023) demonstrated that moxibustion applied at Baihui (GV20) and Dazhui (GV14) significantly improves neurological function, reduces infarct volume, and decreases neuronal death in cerebral I/R injury. These protective effects appear to be mediated by the regulation of ferroptosis‐related metabolic pathways, including GSH metabolism, lipid metabolism, and iron metabolism. The thermal properties of moxibustion may offer distinct advantages in modulating these pathways, providing an additional layer of neuroprotection.

Overall, acupuncture‐related therapies, including traditional acupuncture, EA, and moxibustion, target multiple aspects of ferroptosis, including oxidative stress, iron homeostasis, and lipid peroxidation. These interventions act through distinct yet complementary mechanisms, offering a multifaceted and promising approach to mitigating I/R injury and improving neurological outcomes in IS.

### 4.2 Mechanisms of Acupuncture‐Related Therapies in Modulating Ferroptosis in HS

HS, which includes intracerebral hemorrhage (ICH) and subarachnoid hemorrhage (SAH), is a severe subtype of stroke characterized by high mortality and significant neurological impairment (Magid‐Bernstein et al. 2022). The primary pathophysiology of HS involves the infiltration of blood into the brain parenchyma, where lysed red blood cells release hemoglobin into the interstitial space. Hemoglobin degradation by heme oxygenase generates free iron, which drives oxidative stress and ferroptosis. Ferroptosis exacerbates neuronal damage by promoting lipid peroxidation and disrupting cellular homeostasis (Magid‐Bernstein et al. 2022). Studies have demonstrated that ferroptosis inhibitors can attenuate neuronal death and reduce iron deposition after cerebral hemorrhage, highlighting ferroptosis as a critical therapeutic target in HS (Cao et al. 2021; J. Hu, Cheng, et al. 2024).

MicroRNAs (miRNAs) are emerging as key modulators in the pathogenesis of HS (Kashif et al. 2021). Dysregulation of miRNAs expression has been linked to poor outcomes in HS, making miRNAs‐targeted interventions a promising therapeutic strategy (Sun et al. 2025). Recent studies indicate that acupuncture can modulate miRNAs expression to exert neuroprotective effects. Kong et al. (2021) demonstrated that the “Baihui‐penetrating‐Qubin” acupuncture method significantly reduces neurological deficits, brain edema, inflammation, and ferroptosis in a rat model of ICH. Mechanistically, this effect is mediated by a reduction in miR‐23a‐3p levels and activation of the NRF2 signaling pathway. MiR‐23a‐3p suppression alleviates neuronal degeneration, inflammation, and ferroptosis, while NRF2 activation enhances antioxidant responses and reduces oxidative damage. As a master regulator of cellular antioxidant defenses, NRF2 plays a central role in mitigating ferroptosis by upregulating genes involved in GSH metabolism and lipid peroxidation repair (Yan et al. 2023). These findings suggest that targeting the miR‐23a‐3p/NRF2 pathway through acupuncture may offer a novel therapeutic approach for early brain injury in ICH.

Scalp acupuncture (SA) has also been recognized as an effective therapeutic modality for promoting recovery from neurological impairments following HS (X. Hu et al. 2021). Clinical studies and meta‐analyses have highlighted SA's efficacy in improving motor function and reducing neurological deficits in stroke patients (Y. J. Huang, Huang, et al. 2021). Mechanistic investigations further reveal that SA exerts its neuroprotective effects by activating the p62/Keap1/NRF2 signaling pathway and modulating ferroptosis‐related proteins including ferritin heavy chain 1 (FTH1) and GPX4 (M. Y. Li et al. 2022). Under conditions of oxidative stress, p62 disrupts the interaction between Kelch‐like ECH‐associated protein 1 (Keap1) and NRF2, preventing NRF2 degradation and promoting its nuclear translocation. In the nucleus, NRF2 upregulates the expression of antioxidant and ferroptosis‐inhibitory genes, including GPX4, which reduces lipid peroxidation, and FTH1, which sequesters free iron. These molecular mechanisms suggest that SA may enhance cellular resilience against ferroptosis and oxidative stress, providing a targeted approach to improve outcomes in patients with acute ICH.

EA further expands the therapeutic potential of acupuncture‐related interventions in HS. Q. Chen et al. (2022) reported that EA at Baihui (GV20) and Dazhui (GV14) acupoints significantly reduces neurological severity scores and promotes neuroprotection in a rat model of cerebral hemorrhage. The underlying mechanism involves modulation of iron metabolism, as EA reduces the expression of hepcidin, a key hormone regulating systemic iron homeostasis, as well as ferritin, TF, and TFR proteins. Hepcidin plays a critical role in maintaining iron balance by controlling intestinal iron absorption and cellular iron release (Nemeth and Ganz 2023). By reducing hepcidin levels and iron‐related protein expression, EA alleviates iron overload and oxidative stress, mitigating ferroptosis and its associated neuronal damage. This demonstrates the multifaceted role of EA in regulating iron homeostasis and protecting against ferroptosis in the context of HS.

Overall, acupuncture‐related therapies, including traditional acupuncture, SA, and EA, exhibit significant potential in mitigating ferroptosis and its detrimental effects in HS. These therapies target multiple pathways, including the regulation of miRNAs expression, activation of antioxidant systems, and modulation of iron metabolism. By addressing key pathological processes including oxidative stress, iron overload, and lipid peroxidation, acupuncture‐related interventions provide a multifaceted approach to reducing neuronal damage and improving clinical outcomes in HS.

## 5 Anti‐Ferroptosis Effect of Acupuncture‐Related Therapies in Age‐Related NDDs

NDDs, such as Alzheimer's disease (AD) and Parkinson's disease (PD), represent a heterogeneous group of disorders characterized by the progressive degeneration and loss of neurons (Q. Jiang et al. 2025). Despite distinct clinical presentations and pathological features, these conditions share common underlying mechanisms, including oxidative stress (Kim et al. 2024), neuroinflammation (Giri et al. 2024), and metal dysregulation (Y. Y. Zhang, Li, et al. 2023). Currently, small‐molecule drugs remain the primary clinical treatments for NDDs (W. Liu, Wang, et al. 2022; Miller and Blanco 2021). However, they are largely palliative and fail to halt or reverse neurodegeneration, leaving an urgent need for alternative therapeutic approaches.

Iron accumulation has emerged as a shared pathological hallmark across several age‐related NDDs (L. Chen et al. 2025). Excess iron is frequently observed in affected brain regions, such as the substantia nigra in PD (Wen et al. 2025) and the hippocampus in AD (J. Zhou, Wearn, et al. 2024), and is strongly correlated with disease progression. Advanced imaging techniques, such as quantitative susceptibility mapping, have facilitated the precise tracking of iron deposition in vivo, further supporting the role of iron dysregulation in neurodegeneration (Fushimi et al. 2024). Furthermore, genetic studies have identified that mutations in genes responsible for iron metabolism are linked to adult‐onset neurodegenerative disorders (Alvarez Jerez et al. 2023). These findings underscore the critical role of iron metabolism in the pathogenesis of NDDs and suggest that targeting iron dysregulation could be a promising therapeutic strategy.

Ferroptosis has been implicated as a key mechanism linking iron accumulation to neurodegeneration in NDDs (Alrouji et al. 2024). Pathological iron overload enhances the production of ROS via Fenton reactions, exacerbating lipid peroxidation and triggering ferroptosis. This cascade of events contributes to neuronal loss and accelerates disease progression. While brain‐penetrant iron chelators have been explored as a therapeutic strategy to reduce iron overload, their clinical application has been limited due to mixed efficacy and potential adverse effects (Singh et al. 2019). Iron chelators can disrupt essential iron‐dependent physiological processes, such as mitochondrial respiration and neurotransmitter synthesis, highlighting the challenge of balancing therapeutic benefits with safety. Consequently, there is an urgent need to develop alternative therapies that effectively modulate iron metabolism and prevent ferroptosis without disrupting normal cellular functions.

### 5.1 Mechanisms of Acupuncture‐Related Therapies in Modulating Ferroptosis in AD

AD is characterized by the progressive degeneration of neurons in the central nervous system, with hallmark pathological features including β‐amyloid (Aβ) plaques, neurofibrillary tangles (NFTs) composed of hyperphosphorylated tau, and widespread neuronal loss (Roda et al. 2022). In addition to these features, other pathological processes such as oxidative stress (Spina et al. 2025), neuroinflammation (Lee and Chang 2025), apoptosis (Kumari et al. 2023), autophagy dysregulation (W. Zhang, Xu, et al. 2022), and metal dyshomeostasis (Feng et al. 2023) are strongly implicated in AD progression. Notably, intracellular iron accumulation has been observed prior to the formation of Aβ plaques and NFTs, suggesting that iron dysregulation may play a causative role in neuronal damage (Derry et al. 2020). Excess iron contributes to the generation of ROS via Fenton reactions, exacerbating oxidative stress and lipid peroxidation. This cascade ultimately triggers ferroptosis, which has been identified as a significant contributor to neuronal loss in AD.

Acupuncture‐related therapies have shown significant potential in addressing the pathological mechanisms of AD, particularly by targeting ferroptosis. Evidence‐based studies suggest that acupuncture may outperform pharmacological treatments in improving cognitive function and enhancing daily living activities in AD patients (Q. Huang et al. 2019). Mechanistically, EA has been found to activate the p62/Keap1/NRF2 signaling pathway, a critical regulator of cellular antioxidant defenses (Y. Chen et al. 2024). Specifically, EA upregulates the expression of p62, a scaffold protein that promotes the dissociation of Keap1 from NRF2 (L. Fan et al. 2018). This dissociation enables NRF2 to translocate into the nucleus, where it drives the transcription of genes involved in antioxidant responses and ferroptosis regulation, including GPX4 and FTH1. GPX4 is an essential antioxidant enzyme that mitigates lipid hydroperoxides, protecting neurons from ferroptosis, while FTH1 sequesters excess free iron, reducing its availability for ROS generation. These findings highlight the pivotal role of the NRF2 signaling pathway in alleviating oxidative damage and preventing ferroptosis, underscoring EA's potential as a neuroprotective therapy in AD.

In addition to modulating the p62/Keap1/NRF2 pathway, EA has been shown to regulate other ferroptosis‐related factors that contribute to its neuroprotective effects. A recent study has demonstrated that EA enhances spatial learning and memory abilities in murine models of AD while mitigating neuronal cell death and inhibiting lipid peroxidation (Y. T. Li et al. 2023). These beneficial outcomes are attributed to the upregulation of GPX4 and the downregulation of prostaglandin‐endoperoxide synthase 2 (PTGS2), also known as COX‐2. PTGS2 is an enzyme involved in prostaglandin biosynthesis, which exacerbates inflammation and oxidative stress (Moussa and Dayoub 2023). By simultaneously enhancing GPX4 activity and suppressing PTGS2 expression, EA effectively inhibits ferroptosis, reduces oxidative stress and neuroinflammation, and further contributes to neuronal protection in AD.

Notably, both studies employed the “Tongdu Qishen” needle technique, which specifically targets the Baihui (GV20), Yintang (EX‐HN3), and Shuigou (GV26) acupoints. These acupoints are located along the Du Meridian, traditionally believed to “enter the brain” and regulate the body's yang energy. The strategic selection of these acupoints is thought to enhance the therapeutic efficacy of EA by modulating the Du Meridian, thereby improving cognitive function and providing neuroprotection against oxidative stress and ferroptosis.

Collectively, acupuncture‐related therapies, particularly EA, offer a multifaceted approach to addressing the pathological mechanisms of AD. By activating the p62/Keap1/NRF2 signaling pathway, modulating ferroptosis‐related factors such as GPX4 and PTGS2, and targeting specific acupoints along the Du Meridian, EA demonstrates significant potential as a neuroprotective therapy. These findings provide a deeper understanding of how acupuncture‐related interventions can alleviate oxidative stress, inhibit ferroptosis, and ultimately improve cognitive outcomes in AD.

### 5.2 Mechanisms of Acupuncture‐Related Therapies in Modulating Ferroptosis in PD

PD represents another significant degenerative disorder of the central nervous system. Approximately 90% of PD cases are sporadic, influenced by various risk factors, while the remaining 10% are familial, primarily linked to mutations in the α‐synuclein gene (Mochizuki 2024; Moussa and Dayoub 2023). The disease is characterized by dopaminergic neuron degeneration in the substantia nigra pars compacta (SNpc), resulting in striatal dopamine deficiency, neuromelanin loss, and Lewy body formation. Pathological iron accumulation in the SNpc correlates with neuronal degeneration and drives oxidative stress through lipid peroxidation (Yao et al. 2024). Importantly, α‐synuclein aggregation and lipid peroxidation mutually reinforce each other, while iron deposition amplifies this degenerative cascade, collectively contributing to PD progression (Ding et al. 2023).

Targeting ferroptosis has emerged as a promising therapeutic strategy in the treatment of PD (W. Wang et al. 2025). Inhibitors of ferroptosis have demonstrated potential in reducing neuronal death and mitigating iron accumulation in animal models of PD (X. Jiang et al. 2023). Nevertheless, existing therapeutic options for PD remain limited. Current pharmacotherapies primarily manage symptoms without halting or decelerating the degeneration of dopaminergic neurons and are frequently associated with adverse effects following long‐term use (Mao et al. 2020). This limitation has spurred interest in exploring alternative therapeutic modalities, notably TCM techniques such as acupuncture‐related therapies, which demonstrate potential in mitigating the neurodegenerative processes characteristic of PD.

Empirical studies suggest that acupuncture may exert therapeutic effects on PD by enhancing cerebral blood flow, augmenting neural activity, reducing inflammatory responses, improving gastrointestinal function, and safeguarding dopaminergic neurons, thereby ameliorating both motor and non‐motor symptoms associated with the disease (J. Q. Fan et al. 2022). A 2023 study specifically demonstrated that EA applied to the Baihui (GV20), Quchi (LI11), and Zusanli (ST36) acupoints exerts neuroprotective effects on dopaminergic neurons in the substantia nigra of PD model mice (Ma et al. 2023). Mechanistically, this protection appears to be mediated through EA's ability to regulate oxidative stress pathways and inhibit ferroptosis‐induced apoptosis. The findings collectively highlight acupuncture's potential to modulate key pathological processes in PD through ferroptosis.

Moxibustion, another TCM technique, has garnered increasing attention for its potential in PD therapy. Recent studies have demonstrated that moxibustion exerts significant neuroprotective effects by targeting ferroptosis, offering a novel approach to PD treatment. Initial research by Lu et al. (2019) revealed that moxibustion increased the levels of key proteins such as GPX4 and FTH1 in PD rats, inhibited the progression of ferroptosis, and enhanced the number of tyrosine hydroxylase (TH)‐positive neurons in the substantia nigra. As TH is a critical rate‐limiting enzyme in dopamine synthesis and a marker for midbrain neurons, its decline is closely associated with dopamine deficiency and the manifestation of PD symptoms (Yi et al. 2024). These findings highlight the potential of moxibustion as an early intervention strategy to protect dopaminergic neurons and delay disease progression.

Building on these findings, subsequent research further explored the link between ferroptosis and dopaminergic neuron injury in PD models. It was demonstrated that moxibustion effectively alleviates neuronal damage by suppressing ferroptosis, thereby reinforcing its neuroprotective role (Z. Huang, Si, et al. 2021). These results not only validated earlier observations but also highlighted moxibustion's therapeutic potential in mitigating the neurodegenerative processes of PD and improving clinical outcomes. More recently, the research team discovered that moxibustion could improve motor dysfunction in PD mice, further expanding its therapeutic implications (D. Gao et al. 2024). This effect was attributed to moxibustion's ability to modulate key ferroptosis‐related pathways, including downregulating sorting nexin 5 (SNX5), promoting GSH synthesis, reducing Fe^2^⁺ accumulation and malondialdehyde (MDA) levels, restoring the GSH/glutathione disulfide (GSSG) ratio, and upregulating the mRNA and protein expression of GPX4 and FTH1 in the corpus striatum. These multifaceted effects position moxibustion as a promising adjunctive therapy for PD, providing a strong foundation for further clinical investigation.

Collectively, acupuncture‐related therapies such as EA and moxibustion have demonstrated significant potential in modulating ferroptosis, a critical pathological process in PD. By simultaneously targeting interconnected mechanisms such as iron homeostasis, oxidative stress, and dopaminergic neuron survival, these therapies offer a novel, multi‐target approach to addressing the neurodegeneration associated with PD.

## 6 Future Perspectives and Challenges

Acupuncture‐related therapies targeting ferroptosis in neurological disorders represent a promising integration of TCM with modern neurotherapeutics, offering broad prospects for future development. However, to ensure their safe and effective translation into clinical practice, several key challenges must be addressed, particularly regarding mechanistic understanding, standardization, efficacy specificity, therapeutic integration, and long‐term safety.

### 6.1 Mechanistic Understanding: From Qualitative Descriptions to Quantitative Insights

Emerging evidence suggests that acupuncture‐related therapies modulate ferroptosis through enhancing GPX4 expression, suppressing lipid ROS accumulation, and regulating iron metabolism. Despite these findings, the precise molecular mechanisms remain incompletely characterized. Many studies report qualitative or semiquantitative changes in ferroptosis‐associated markers—such as elevated GPX4 levels or reduced MDA concentrations—but lack standardized experimental designs and consistent outcome reporting, limiting cross‐study comparability.

Furthermore, although alterations in upstream regulatory pathways such as mTOR/SREBP1 and p62/Keap1/Nrf2 have been described, most studies do not provide quantitative assessments—such as fold changes, temporal dynamics, or dose‐response relationships—which hinders our ability to evaluate the magnitude, specificity, and reproducibility of acupuncture‐induced effects on ferroptosis.

To address these limitations, future research should emphasize methodological rigor by employing quantitative biochemical assays with absolute measurements (e.g., ELISA, mass spectrometry‐based proteomics), systematically reporting effect sizes to allow cross‐study comparisons, and conducting time‐course and dose‐response experiments to define the temporal and intensity‐dependent regulation of ferroptotic signaling. In addition, integrating multi‐omics approaches—including single‐cell transcriptomics, proteomics, and lipidomics—will enable a more comprehensive dissection of complex signaling networks and help identify key regulatory nodes affected by acupuncture‐related therapies. Such advances will be critical for transitioning from descriptive observations to a mechanistically grounded and quantitatively supported understanding of acupuncture‐related therapies’ role in modulating ferroptosis.

### 6.2 Standardization: A Prerequisite for Reproducibility and Clinical Translation

A major barrier to advancing this field is the lack of standardized protocols across preclinical and clinical studies. Significant variability exists in acupoint selection, stimulation parameters (e.g., depth, frequency, duration, intensity), treatment regimens, and animal models (including species, strain, age, and disease induction methods). These inconsistencies compromise data comparability and hinder scientific reproducibility. Moreover, there is currently no consensus on validated ferroptosis‐related biomarkers—such as GPX4, FTH1, ACSL4, or MDA—for assessing therapeutic outcomes. The absence of standardized metrics further complicates objective evaluation and meta‐analysis.

To enhance methodological rigor, we strongly recommend the adoption of consensus‐based acupuncture intervention frameworks tailored for preclinical models, along with the implementation of standardized outcome measures reflecting core ferroptotic processes. In addition, adherence to established reporting guidelines—such as the ARRIVE (Animal Research: Reporting of In Vivo Experiments) guidelines—is essential to promote transparency and improve the quality of published research. These efforts are critical for establishing acupuncture‐related therapies as reliable, evidence‐based interventions targeting ferroptosis.

### 6.3 Efficacy Specificity and Disease Context Dependency: Shared Mechanisms, Divergent Outcomes

Although multiple studies indicate that acupuncture‐related therapies exert anti‐ferroptotic effects across various neurological diseases, suggesting potential shared neuroprotective mechanisms, their specific therapeutic effects are significantly influenced by disease context.

In models of IS, HS, PD, and AD, acupuncture has been shown to effectively regulate core ferroptosis regulators such as GPX4, ACSL4, FTH1, and lipid ROS. This suggests that acupuncture may act on evolutionarily conserved redox homeostasis and iron metabolism pathways—pathways commonly dysregulated in various neurological diseases.

However, under different disease backgrounds, the functional importance and therapeutic responsiveness of these mechanisms vary significantly. For example, in PD models, acupuncture primarily acts on dopaminergic neurons in the substantia nigra, where its neuroprotective effect is closely associated with mitochondrial dysfunction and α‐synuclein pathology, showing high regional specificity. In contrast, in AD models, its mechanism is more indirect, mainly involving the regulation of glial activation and Aβ‐induced oxidative stress, which secondarily affects ferroptosis signaling.

This dual characteristic—shared mechanisms with disease‐specific outcomes—suggests that acupuncture‐related therapies can serve as a foundational strategy for targeting ferroptosis across diseases. However, in practical applications, individualized adjustments must be made according to the specific pathophysiological characteristics of each disease to achieve optimal therapeutic effects.

### 6.4 Integration With Conventional Therapies: Toward Multimodal Neuroprotection

An emerging opportunity lies in integrating acupuncture‐related therapies with conventional pharmacological treatments to achieve synergistic neuroprotection. Combining acupuncture with known ferroptosis inhibitors—such as ferrostatin‐1, liproxstatin‐1, or iron chelators like DFO—may offer dual protection by targeting multiple nodes in the ferroptosis pathway. Furthermore, multimodal strategies that combine acupuncture‐related therapies with drugs targeting oxidative stress, mitochondrial dysfunction, or neuroinflammation may lead to improved therapeutic outcomes in both motor and non‐motor symptoms of neurological disorders. Notably, acupuncture‐related therapies may enhance the efficacy of dopaminergic treatments in PD by reducing oxidative burden and stabilizing cellular redox homeostasis—potentially enabling lower drug dosages and reduced side effects, especially in elderly patients.

Future research should prioritize several actionable directions: preclinical studies should evaluate combination therapies in well‐established models of stroke and age‐related NDDs, focusing on mechanistic synergy, temporal coordination, and dose optimization; early‐phase clinical trials should assess the safety, tolerability, and preliminary efficacy of integrative approaches, especially in early‐to‐moderate‐stage patients; and biomarker‐driven monitoring should be implemented to track changes in ferroptosis‐related markers and downstream neuroinflammatory indicators, thereby providing objective evidence of targeted modulation. Such integrative strategies align with the growing shift toward personalized and precision medicine, offering a promising path forward in the treatment of neurological disorders.

### 6.5 Long‐Term Safety Considerations: Balancing Protection and Risk

While short‐term inhibition of ferroptosis via acupuncture‐related therapies may confer neuroprotection, the long‐term consequences of modulating this evolutionarily conserved cell death pathway remain poorly understood. Ferroptosis plays critical roles in tissue homeostasis, immune surveillance, and tumor suppression. Chronic or systemic modulation of this pathway—whether through pharmacological agents or non‐pharmacological interventions like acupuncture‐related therapies—could lead to unintended biological consequences, including impaired cellular quality control, altered iron metabolism, disruption of antioxidant defenses, and interference with antitumor immunity.

Therefore, future research must include biomarker‐guided monitoring of iron status (e.g., serum ferritin, hepcidin, TfR1), oxidative stress markers (e.g., MDA, 4‐HNE, GSH/GSSG ratio), and organ function tests to detect potential systemic side effects. To mitigate risks, it is also essential to emphasize spatial and temporal specificity in acupuncture application, ensuring localized rather than systemic modulation of ferroptosis. Longitudinal studies are needed to assess the safety profile of repeated acupuncture interventions over extended periods, particularly in vulnerable populations such as the elderly or those with comorbid conditions.

### 6.6 Supporting Clinical Translation: From Bench to Bedside

Translating acupuncture‐based anti‐ferroptotic strategies from preclinical models to human applications presents additional challenges. Species‐specific differences in ferroptosis‐related pathways may limit the predictive validity of animal models, while patient heterogeneity—including age, disease stage, comorbidities, and medication use—can influence treatment response. Additionally, the lack of noninvasive tools for monitoring ferroptosis in humans remains a major obstacle to evaluating therapeutic efficacy in real time.

To bridge this translational gap, we advocate for incorporating ferroptosis‐related biomarkers into mechanism‐informed clinical trial designs and utilizing advanced neuroimaging techniques—such as functional magnetic resonance imaging (fMRI), positron emission tomography (PET), and diffusion tensor imaging (DTI)—to monitor dynamic changes in brain activity and metabolic status during acupuncture‐related interventions. Conducting large‐scale, multicenter clinical trials with extended follow‐up will be essential to confirm long‐term safety and efficacy. Only through rigorous and systematic investigation can acupuncture‐based therapies targeting ferroptosis gain broader acceptance in mainstream neuroscience and clinical neurology.

## 7 Conclusion

The growing body of evidence underscores the critical role of ferroptosis in the pathogenesis of neurological disorders. As a regulated form of cell death driven by iron‐dependent lipid peroxidation, ferroptosis contributes to neuronal loss, synaptic dysfunction, and neuroinflammation—key pathological features across a spectrum of neurological disorders. Despite advances in understanding its molecular underpinnings, effective therapeutic strategies targeting ferroptosis remain limited within conventional pharmacology.

In this context, acupuncture‐related therapies have emerged as promising complementary interventions that modulate multiple nodes within the ferroptotic cascade. Accumulating experimental data demonstrate that these therapies exert robust anti‐ferroptotic effects through mechanisms involving iron homeostasis regulation, enhancement of antioxidant defenses, suppression of lipid peroxidation, and mitigation of neuroinflammatory responses. These findings provide a compelling biological rationale for integrating acupuncture‐related therapies into multimodal neuroprotective strategies aimed at preserving neuronal integrity and function.

Moreover, preclinical studies have highlighted the therapeutic potential of acupuncture‐related therapies in both ischemic and HS models, as well as in age‐related neurodegenerative diseases. The ability of acupuncture‐related therapies to synergize with known ferroptosis inhibitors or conventional pharmacotherapies further supports its translational value in developing precision‐based treatment paradigms.

However, significant challenges remain. The heterogeneity of acupuncture protocols, variability in animal models, and limited mechanistic depth in clinical settings underscore the need for standardized methodologies and biomarker‐driven research. Future investigations should prioritize translational studies that bridge mechanistic insights with clinically relevant outcomes, particularly in early‐stage patients where disease‐modifying interventions may yield the greatest benefit.

In conclusion, the integration of acupuncture‐related therapies with modern ferroptosis biology not only expands the therapeutic armamentarium against neurological disorders but also exemplifies a successful convergence of empirical medical traditions with contemporary neuroscience. By targeting fundamental cellular processes with high biological relevance, acupuncture‐related therapies offer a novel and promising avenue for advancing neuroprotective medicine in the era of personalized healthcare.

## Author Contributions

**Haiyan Sun**: conceptualization, writing–original draft, writing–review and editing, investigation, validation, supervision, project administration. **Haiqing Qian**: conceptualization, investigation, validation, supervision, project administration, writing–original draft, writing–review and editing. **Heng Qian**: writing–review and editing, software. **Min Qian**: project administration, supervision, conceptualization, writing–review and editing.

## Ethics Statement

The authors have nothing to report.

## Conflicts of Interest

The authors declare no conflicts of interest.

## Peer Review

The peer review history for this article is available at https://publons.com/publon/10.1002/brb3.70827

## Data Availability

The authors have nothing to report.
